# *Brucella canis* discospondylitis in 33 dogs

**DOI:** 10.3389/fvets.2022.1043610

**Published:** 2022-11-04

**Authors:** Christina Long, Elisabeth Burgers, Christina Copple, Laura Stainback, Rebecca A. Packer, Kelli Kopf, Jessica Schmidt, Samantha Emch, Rebecca Windsor

**Affiliations:** ^1^Wheat Ridge Animal Hospital, Wheat Ridge, CO, United States; ^2^Diagnostic Imaging, P.C, Denver, CO, United States; ^3^Veterinary Neurology Center, Phoenix, AZ, United States; ^4^College of Veterinary Medicine and Biomedical Sciences, Colorado State University, Fort Collins, CO, United States; ^5^VCA Alameda East Veterinary Hospital, Denver, CO, United States

**Keywords:** infection, vertebra, osteomyelitis, discitis, brucellosis

## Abstract

**Objective:**

To describe the clinical and imaging findings of 33 dogs with *Brucella canis* discospondylitis (BDS).

**Animals:**

33 client owned dogs from four veterinary specialty hospitals within Colorado and Arizona with at least one positive *B. canis* test and spinal diagnostic imaging.

**Procedures:**

Retrospective review of signalment, physical and neurological examination findings, laboratory results, *B. canis* serology, and diagnostic imaging of 33 dogs with BDS. All imaging was reviewed by a board-certified veterinary neurologist. Radiographs were reviewed by a board-certified veterinary radiologist blinded to MRI and CT findings.

**Results:**

31/33 (94%) dogs were <5 years old (median = 2.5 years, mean = 2.9 years, range 0.5–10 years). 21/29 (72%) dogs had signs of nonspecific pain, spinal pain, or lameness for >3 months (median = 6 months, mean = 8.2 months, range 5 days−4 years). Fever was seen in only 4/28 (14%) dogs. Multifocal lesions were evident on radiographs in 21/29 (72%) dogs and MRI in 12/18 (67%) dogs. Smooth, round, central end-plate lysis, defined as “hole punch” lesions, were identified radiographically in 25/29 (86%) dogs. Vertebral physitis or spondylitis without discitis was evident on MRI in 7/18 (39%) dogs.

**Clinical relevance:**

Dogs with BDS typically present at a young age with a long duration of clinical signs. Identification of radiographic “hole punch” lesions and MRI evidence of vertebral physitis, spondylitis, and paravertebral inflammation without discitis should increase suspicion for BDS. BDS may be increasing in frequency in the southwestern United States, and dogs with signs of chronic spinal pain and/or lameness should be screened for *B. canis*.

## Introduction

Discospondylitis (DS) is defined as infection of the intervertebral disc and adjacent endplate, which contains a cartilaginous layer that fuses with the intervertebral disc and a bony endplate that attaches to the vertebrae. Discospondylitis is most commonly bacterial, with *Staphylococcus spp* being the most frequently isolated organism (1–5). *Brucella canis (B. canis)*, a gram-negative coccobacillus, is the primary cause of canine brucellosis and can result in DS (6, 7). Brucellosis is typically transmitted venereally or through oronasal contact with vaginal discharges during estrus or abortion, resulting in epidemic outbreaks in stray and breeding populations (6–14). *B. canis* has a worldwide distribution and within the United States is reported most frequently in the southeastern and midwestern states (6–11, 15).

Risk for DS increases with age, with the greatest risk in dogs >10 years old (5, 16–18). DS is reported most commonly in large-breed dogs with a male gender predisposition (1, 3–5, 11, 16). Clinical signs include spinal pain in the majority of dogs (2, 4, 19) and fever in approximately 30% of dogs (1, 4, 20). The highest percentage of lesions affect primarily the intervertebral space of the lumbosacral junction followed by the thoracolumbar segment (1–3).

Diagnosis of DS is made via a combination of diagnostic imaging and identification the causative organism from blood, urine, and occasionally the intervertebral disc (1, 2, 4, 21). There is a reported *B. canis* isolation rate of 8.9–10% in dogs with DS (11, 22), which may be erroneously low due to intermittent bacteremia and organism shedding (7, 23, 24) and previous use of antimicrobial therapy (11, 25). *Brucella* diagnosis via culture or PCR are gold standard, however isolation can be limited by reduced organism detection in the first 3–4 weeks post infection and intermittent shedding in chronic cases (6, 26). The rapid slide agglutination test (RSAT) detects antibodies against surface antigens of *Brucella canis* and is generally sensitive but can be poorly specific due to cross reactivity with other bacteria (6, 26). Addition of 2-mercaptoethanol (2-ME RSAT) improves specificity to 100% (24). Agar-gel immunodiffusion (AGID) uses cytoplasmic antigens conserved only in organisms belonging to the genus *Brucella*, resulting in high specificity (6, 26).

There are limited reports of clinical findings and diagnostic imaging results in dogs with *Brucella canis* discospondylitis (BDS) (11, 23). Transfer of stray rescue dogs from Texas, New Mexico, and Mexico in recent years has resulted in a perceived increase in BDS by neurologists throughout Arizona and Colorado. The purpose of this study was to describe the signalment, clinical features, lesion location, and imaging characteristics in a series of dogs with BDS in Colorado and Arizona compared to previously reported clinical and imaging findings for DS secondary to *B. canis* and other bacterial organisms.

## Materials and methods

### Case selection and medical record review

Medical records and diagnostic imaging were reviewed for 33 dogs with BDS from four veterinary hospitals in Colorado and Arizona. Dogs were included if *Brucella canis* alone was cultured from urine or blood and/or a positive result was obtained on 2ME-RSAT and/or cytoplasmic antigen AGID which have a reported 100% specificity (24). While specificity of Indirect Fluorescent Antibody (IFA) is unknown, dogs with IFA greater than or equal to 1:200 were included as this correlates with a high (at least 80%) isolation rate of *Brucella* and is suggestive of active infection (10, 24). Dogs with positive *B. canis* diagnostics which cultured other organisms were excluded given potential for cross reactivity for some bacterial species. Dogs positive for *Brucella canis* with suspect DS that lacked spinal imaging were excluded. The duration of clinical signs was considered chronic if the clinical signs had been present for 3 months or longer (27). Breed size was categorized into small (<10 kg), medium (10-<25 kg), large (25-<40 kg), and giant (≥40 kg) (28). Dogs were considered febrile if rectal body temperature was greater than 102.5 degrees Fahrenheit (F), or 39.2 degrees Celsius (C) (29). CBC and biochemistry panel abnormalities were recorded. Not all data points were available for every dog which is reflected in the total number of dogs detailed in the results section. Documentation of long-term treatment and outcome was sporadic within the medical records given the recommendation for euthanasia by the Colorado state department and lack of follow-up by many owners, therefore outcome data was excluded from the results. Statistics were performed using Microsoft Excel version 16.4.

### Diagnostic imaging

Imaging modality type, location within the spinal column, and sequence acquisition was determined by clinician preference based on neurological examination abnormalities, image modality availability, and cost to client. Partial vertebral segment imaging was performed in dogs where neuroanatomic localization suggested a focal lesion. The majority of dogs had screening studies including multiple lateral views, and a single, appropriately exposed and positioned, lateral spinal radiograph was considered acceptable for inclusion in the study. Radiographic findings considered most consistent with discospondylitis included vertebral endplate and/or body lysis (1, 2, 5, 30). Images were acquired on various 1.5T MRI machines depending on institution (Siemens MAGNETOM Symphony, General Electric Signa LX, General Electric Signa HDxt). MRI inclusion criteria included at a minimum sagittal short-tau inversion (STIR) sequences, however T2W sagittal and transverse imaging and T1-weighted (T1W-pre) and T1W post-contrast (T1W-post) sequences were available in many dogs. CT studies (Toshiba Aquilion 64-slice helical CT, General Electric 8-slice helical CT) included pre-contrast bone and soft tissue windows with transverse imaging and sagittal and dorsal reconstructions. Diagnostic imaging was reviewed by a board-certified neurologist and diagnostic imaging intern and all lesions were tallied. Radiographs were also reviewed by a board-certified veterinary radiologist who was blinded to MRI and CT findings to better evaluate the utility of radiographs to detect BDS lesions.

## Results

### Signalment and history

Data describing signalment, origin of adoption, and duration of clinical signs are listed in Supplementary Table 1. Geographical origin was available for 23 dogs and included Texas (*n* = 7), Arizona (*n* = 6), New Mexico (*n* = 4), Colorado (*n* = 2), Mexico (*n* = 2), Nebraska (*n* = 1), and a Native American reservation of an unknown location (*n* = 1). Two BDS littermates originating from Texas were from the same litter (adopted by different families). Nine dogs lived in Arizona and 24 dogs lived in Colorado at the time of diagnosis.

The duration of clinical signs prior to presentation ranged from 5 days to 4 years (median = 6 months, mean = 8.2 months), and 21/29 (72%) dogs had chronic signs of 3 months or longer. Three dogs that had clinical signs for 1–2 weeks (and documented as such in the duration of signs) had previous episodes of spinal pain in the previous year. Fifteen dogs showed signs of nonspecific pain, spinal pain, or lameness since being adopted as puppies.

The BDS population included 22 (67%) males and 11 (33%) females. All dogs were neutered except for one 9-month-old intact female. Breeds included 8 purebred or mixed German Shepherd Dogs, 5 purebred or mixed Laborador Retrievers, 2 purebred or mixed Australian Shepherds, 2 Border Collie mixes, 2 Chow Chow mixes, 1 Bernese Mountain Dog, 1 Siberian Husky mix, 1 Staffordshire Terrier Mix, and 1 Australian Cattle Dog mix. There were 10 other mixed breed dogs of unspecified breed.

Size distribution included 1 (3%) small breed dog, 11 (33%) medium breed dogs, 18 (55%) large breed dogs, and 3 (9%) giant breed dogs (body weight median = 27 kg, mean = 27.5 kg, range 8.65–50.5 kg). The majority of dogs were young with 30/33 (91%) less than 5 years of age and 23/33 (70%) 3 years of age or less (median = 2.5 years, mean = 2.9 years, range 0.5–10 years).

### Physical exam abnormalities

Complete physical, orthopedic, and neurological exams were performed on all dogs. Body temperatures were available for 28 dogs at the time of diagnosis (median = 101.5 F/38.6 C, mean = 101.5 F/38.6 C, range 99.4 F/37.4–103.7 F/39.8 C); only 4/28 (14%) dogs were febrile (T > 102.5 F/39.2 C). Spinal pain was the most common clinical sign and was recorded as cervical (*n* = 9), thoracolumbar (*n* = 1), lumbosacral (*n* = 3) or multifocal (*n* = 7). Non-specific pain (*n* = 6) and coxofemoral pain (*n* = 2) were also noted. Lameness was described as unilateral thoracic limb (*n* = 3), bilateral thoracic limb (*n* = 2), unilateral pelvic limb (*n* = 1) or bilateral pelvic limb (*n* = 2). Signs of potential systemic infection included uveitis in 1 dog, and generalized lymphadenopathy in 1 dog. No other physical or neurological examination abnormalities (i.e. paresis/ataxia, postural reaction deficits, spinal reflex abnormalities) were reported.

### Laboratory abnormalities

CBC was abnormal in 4/18 (22%) dogs and included a mild neutrophilia in 2 dogs, mild monocytopenia in 1 dog, mild lymphopenia in one dog, and mild thrombocytopenia in 1 dog (Supplementary Table 2). The 2 dogs with neutrophilia were normothermic, had no evidence of comorbidities (nor did any dog in this study), and had chronic durations (6 and 30 months). Biochemistry abnormalities were noted 6/17 (35%) dogs; the most frequently noted abnormality was mild hyperglobulinemia (*n* = 3, range for elevated dogs 3.9–4.8 g/dL, normal range 2.2–3.7 g/dL). Other mild changes included elevated ALP (*n* = 2), elevated lipase (*n* = 1), hyperbilirubinemia (*n* = 1), and hyperphosphatemia (*n* = 1).

### Brucella diagnostic testing

Brucellosis diagnostic findings are listed in Supplementary Table 3. Results were indicative of *Brucella canis* on a single test in 9 dogs, two tests in 13 dogs, three tests in 10 dogs, and four tests in 1 dog. Urine culture was positive for *Brucella canis* in 1/16 (6%) dog and negative for all bacteria in 15/16 (94%) dogs. Blood culture was positive for *Brucella canis* in 13/18 (72%) dogs and negative for *Brucella canis* in 5/18 (28%) dogs. Culture of the L7-S1 intervertebral disc was acquired intraoperatively in one dog and showed no growth. *Brucella* 2ME-RSAT (Zoetis D-Tec™ CB) was positive in 6/7 (86%) dogs and inconclusive in 1 dog (with positive AGID), and 2ME-RSAT (Cornell) was positive in 15/15 (100%) dogs. *Brucella canis* IFA was positive in 13/14 dogs (93%) and negative in 1 dog (with positive 2ME-RSAT and *Brucella canis* blood culture). AGID was positive in 19/21 (90%) dogs, suspicious in 1 dog (with positive 2-ME RSAT), and negative in 1 dog (with positive 2-ME RSAT and *Brucella canis* blood culture).

### Fungal testing

*Aspergillosis* antigen (Miravista) was negative in 5/5 dogs. *Coccidiodies* antibody (Miravista) was negative in 8/8 dogs.

### Diagnostic imaging

Imaging included radiographs alone in 15 dogs, radiographs and MRI in 13 dogs, MRI alone in 4 dogs, and radiographs, MRI, and CT in 1 dog. There were 109 lesions identified on radiographs, 74 lesions on MRI, and 8 lesions on CT.

#### Radiograph findings

**Figure 1:**
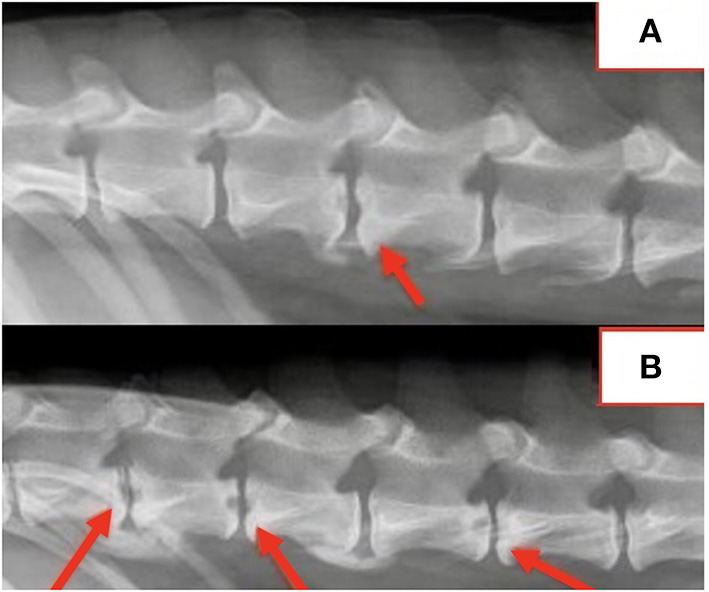
**(A,B)** Lateral radiographs exemplifying typical “hole punch” lesions (red arrows).

Spinal radiographs were reviewed in 29 dogs and included 18 partial spine studies and 11 complete spine studies. Seven (24%) dogs had a single visible radiographic abnormality, 21 (72%) dogs had multiple (≥2) radiographic changes, and one dog had no visible radiographic abnormalities. A total of 109 radiographic abnormalities were identified on spinal radiographs. Many (60/109; 55%) were characterized by a round, well-defined, central lucency that affected both vertebral endplates and were termed “hole punch” lesions, which were apparent in 25/29 (86%) dogs (Figure 1). More diffuse irregular endplate lysis was identified at 36/109 (33%) sites. Other common features included a narrowed intervertebral disc space at 88/109 (81%) sites and sclerotic endplates at 82/109 (75%) sites. There was no correlation between duration of clinical signs and number or type of radiographic abnormality visualized on spinal radiographs.

Location of radiographic changes included 18/109 (17%) cervical, 1/109 (0.9%) cervicothoracic junction, 26/109 (24%) thoracic, 13/109 (12%) thoracolumbar junction, 41/109 (38%) lumbar, and 10/109 (9%) lumbosacral junction. The most common locations noted radiographically included L1-2 in 14/29 (48%) dogs, T13-L1 in 13/29 (45%) dogs, L2-3 in 12/29 (41%) dogs, T12-13 in 10/29 (34%) dogs, and L7-S1 in 10/29 (34%) dogs. Radiographic changes correlated with the location of spinal pain in all dogs. All dogs with multifocal or nonspecific pain had radiographic abnormalities at multiple sites.

#### MRI findings

MRI was available in 18 dogs and included 11 partial spine studies and 7 complete spine studies. Eighteen dogs had STIR sequences, 14 dogs had T2W sequences, 7 dogs had T1W pre-contrast and 14 dogs had T1W post-contrast imaging. A single lesion was noted in 6/18 (33%) dogs and multiple lesions were noted in 12/18 (67%) dogs. Abnormalities were noted at 74 locations and included 16 (22%) cervical, 25 (34%) thoracic, 8 (11%) thoracolumbar junction, 17 (23%) lumbar, and 8 (11%) lumbo-sacral junction. The most common MRI lesion locations were T13-L1 in 8/18 (44%) dogs, L7-S1 in 8/18 (44%) dogs, L1-2 in 6/18 (33%) dogs and L2-3 6/18 (33%) dogs.

**Figure 2:**
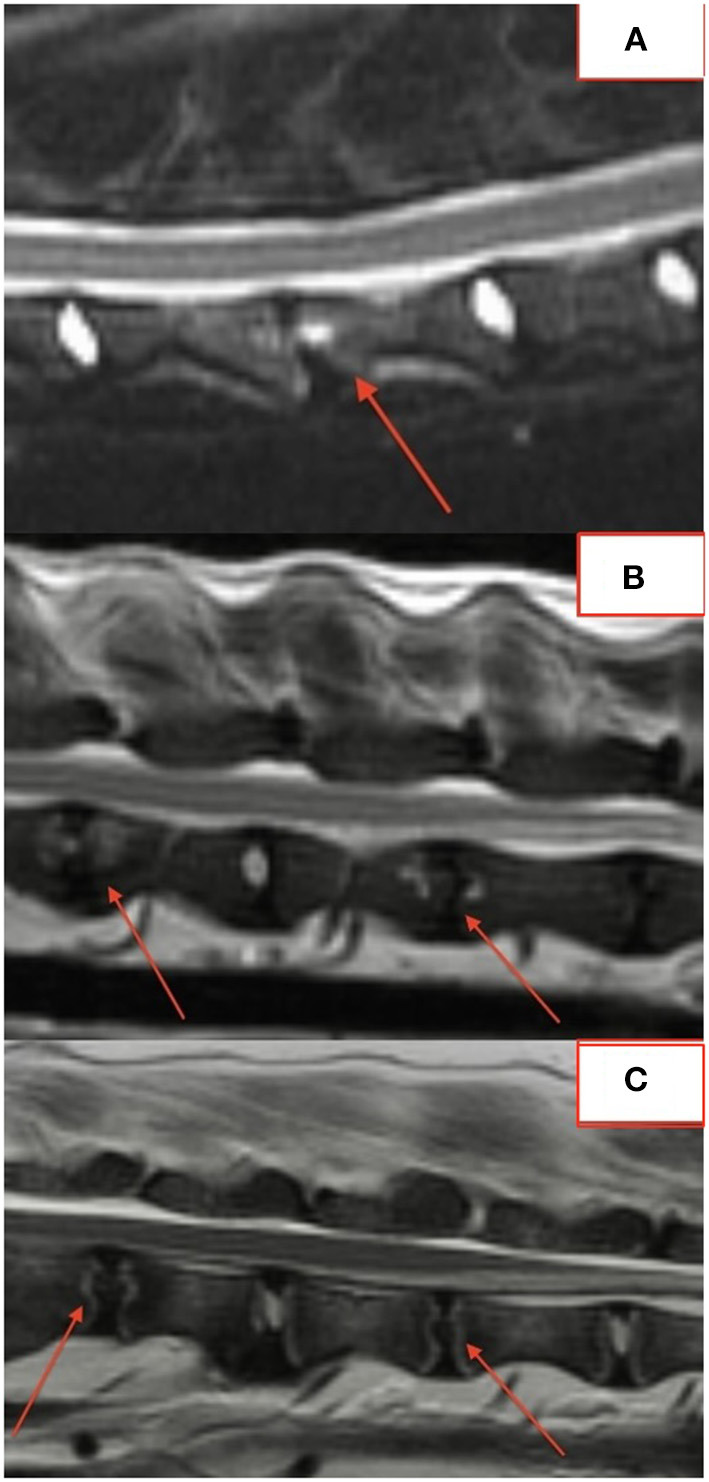
**(A–C)** Examples of “hole punch” lesions on MRI.

The majority of lesions (50/76; 66%) involved the intervertebral disc and vertebral endplates. Focal, well-defined, round lesions affecting the central region of the intervertebral disc and endplate similar to the “hole-punch” lesions noted on radiographs were identified in 7/18 (39%) dogs and comprised 27/74 (36%) lesions (Figure 2).

**Figure 3:**
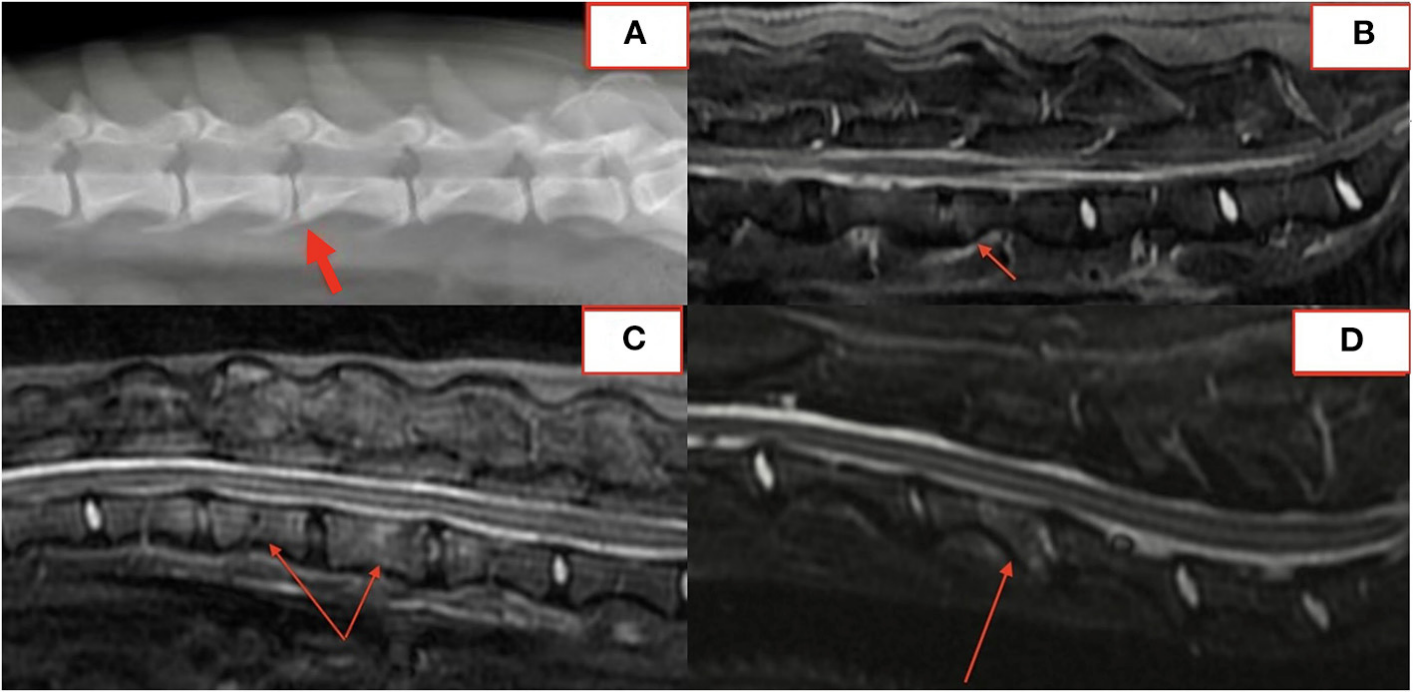
Lateral radiograph **(A)** and STIR MRI **(B)** demonstrating vertebral physitis and osteomyelitis without obvious discitis at L4-5. Lateral STIR imaging at T11-L1 **(C)** and C4-5 **(D)** from two dogs with osteomyelitis without significant intervertebral disc involvement.

**Figure 4:**
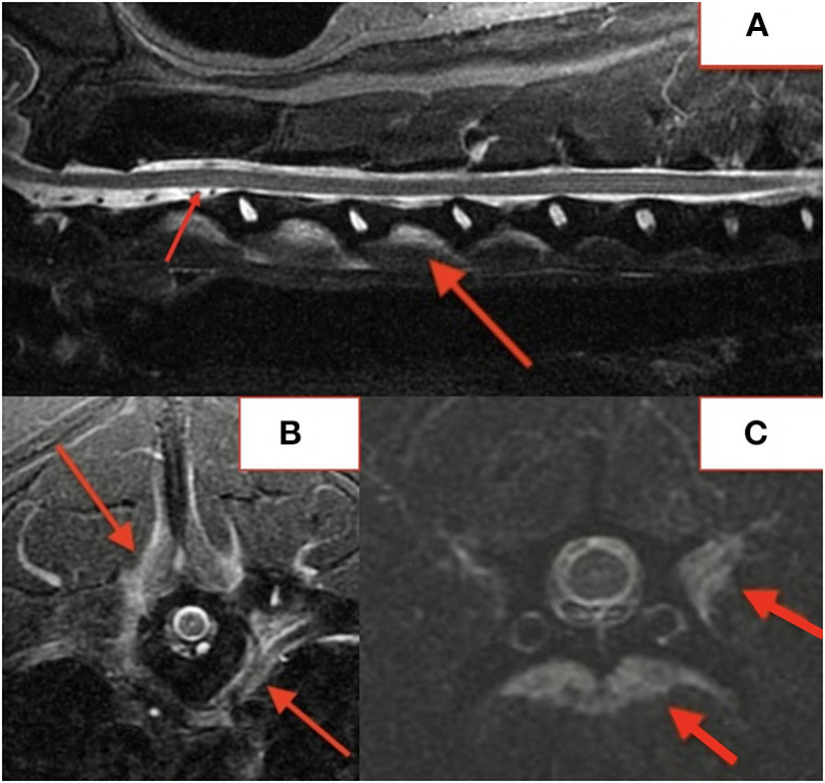
MRI STIR cervical sagittal **(A)** and axial paravertebral lesions at T6 **(B)** and C5 **(C)**.

Several lesions (18/74; 24%) affecting only the bony endplate or vertebral body, determined to represent vertebral physitis or spondylitis without discitis, were apparent in 6/18 (33%) dogs (Figures 3A–D). Abnormalities within the paravertebral soft tissues were noted in 4 dogs. In one dog this paravertebral inflammation was the only abnormality on MRI (Figure 4); radiographs showed no abnormalities in this dog, and this dog was positive on *Brucella* IFA.

STIR, T2W, and T1W-post imaging were available for 18 lesions. Changes to the intervertebral disc space, endplates or vertebral bodies suggestive of DS were most evident on STIR imaging 18/18 (100%) compared to T2W (12/18; 67%) and T1W-post (13/18; 72%) imaging.

#### CT findings

A total of 8 lesions were identified in the single patient with a CT study; these lesions were also seen on the patient's MRI. Lesions were characterized by disc space narrowing (8/8; 100%), irregular end-plate lysis (7/8; 88%), and end-plate sclerosis (7/8; 88%).

#### Comparison of radiographs to MRI

**Figure 5:**
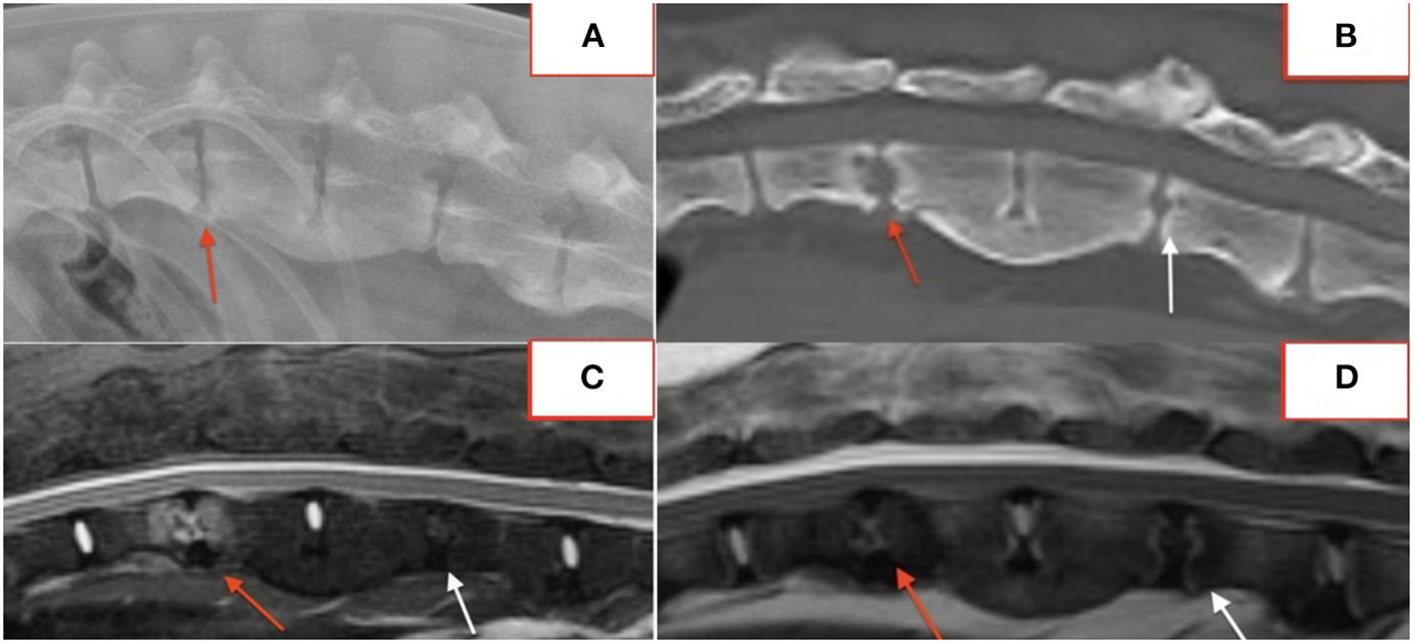
Lateral radiograph **(A)**, CT **(B)**, STIR sagittal MRI **(C)**, T2 sagittal MRI **(D)** demonstrating lesions at T13-L1 and L2-3.

In fifteen patients with radiographs and MRI, 21 of 57 (37%) lesions visible on MRI were not identified on blinded radiograph review by a board-certified radiologist. Six of 21 (29%) unidentified lesions were from a single dog with paravertebral inflammatory changes only, making radiographs alone insufficient to diagnose DS. Of the remaining lesions, 10/21 (48%) affected the vertebral body or osseous endplate with minimal change to the intervertebral disc and 2/21 (10%) were subtle hole-punch lesions on MRI. Figure 5 exemplifies radiographic, CT, and MRI appearance of BDS lesions in one dog.

## Discussion

This study, to the authors' knowledge, represents the largest review of BDS and highlights some important differentiating factors from other causes of DS. The risk for DS is reported to increase with age, with highest incidence in dogs over 10 years (3, 5, 16, 17, 22). The dogs with BDS in this report were younger than previously reported (3, 5, 11, 16, 17, 22), with only 3 (9%) dogs over 5 years old. Mean age at time of presentation was 2.9 years compared to a previously reported mean age range of 5–9 years for DS (5, 16, 31–33) and 4.8 years for BDS (11, 34). The young age at presentation and duration of symptoms since puppyhood in many dogs suggests probable in utero infection, as many surviving puppies develop DS and other manifestations of brucellosis later in life (6). The majority of dogs were mixed breed compared to previous DS reports of purebred dogs (3, 16, 17, 31), which likely reflects the acquisition of many dogs from locations such as Texas, New Mexico, and Mexico where brucellosis within stray mix breed populations has been isolated (6–10, 12, 13, 15, 35). Eight dogs had no travel history outside of Arizona and Colorado, where *B. canis* is not commonly reported (6–11).

The group consisted of predominantly large-breed, male dogs, however the majority were neutered compared to previous reports of BDS where as high as 92% of the males were intact (11). Intact dogs within a stray population are more likely to contract *B. canis* (6, 7, 10, 15, 35), and the reservoir of *B. canis* in the prostate and lymphoid tissues leads to intermittent bacteremia in both neutered and intact males (31, 36). There was only 1 sexually intact dog in this study (one female), suggesting most dogs may have acquired BDS in utero or were neutered after acquiring BDS venereally.

The majority of the dogs (72%) had a chronic presentation with a mean duration of clinical signs of 10.1 months compared to previously reported mean durations of 4 months at the longest with DS (2, 4, 5, 17). *B. canis* is known to persist in tissues and shed intermittently for weeks to years (6, 7, 24, 31, 36). While bacteremia and urine shedding decrease within 2–4 months post-infection (6, 7, 24), blood cultures can be positive for up to 5.5 years after diagnosis (24).

The predominant clinical signs of spinal pain, nonspecific pain, and lameness in dogs with BDS in this study are consistent with other reports of DS (1, 4). Three dogs had signs of systemic inflammation (lymphadenomegaly, uveitis), which may be more specific to brucellosis (6, 15, 24). Few dogs with BDS presented with a fever (4/28, 14%), a lower frequency than dogs with DS from other bacteria (approximately 30%) (1, 4, 20) and humans with brucellosis where cyclical fever is common (37). Of the 4 dogs with a fever, there was no evidence of any other concurrent infectious or inflammatory disease. Durations of clinical signs in these dogs were 5 weeks, 3 months (*n* = 2), and 9 months suggesting no correlation between presence of fever and duration of clinical signs.

CBC and biochemistry panels are often normal in dogs with DS, and laboratory abnormalities suggestive of systemic inflammation were also uncommon in this group (24). Hyperglobulinemia was the most frequent finding which is consistent with other DS reports (20). Only 12.5% (2/16) of dogs exhibited peripheral neutrophilia, which is lower compared to previously reported dogs with DS (20), however leukocytosis is often absent in dogs without concurrent conditions such as endocarditis (4).

A variety of techniques were performed to diagnosis *B. canis* infection. Blood culture is considered gold standard (6) and was positive in 72% of the dogs in this study. However, blood culture is often limited by intermittent bacteremia, reduced detection after the first 1–8 weeks post infection, inadequate culture time (minimum 9 days often required), and previous antibiotic use (7, 38). Urine cultures are typically positive in 25–50% dogs with DS (39). The urine culture rate was low in this group of dogs, potentially because the majority were neutered males. Prostatic secretion is significantly reduced/ceased after orchiectomy (40), and females do not have a reservoir for persistent infection in the urogenital tract (7). Free catch or urinary catheterization may have increased *B. canis* detection in males due to prostatic shedding (6). Intervertebral disc culture may have had a higher sensitivity than blood and urine culture if performed in a larger number of dogs, however intervertebral disc cultures are challenging to obtain and even the intraoperatively acquired intervertebral disc culture showed no growth in one dog in this study (1, 21, 38).

Several *B. canis* serology tests were utilized. 2-ME RSAT had the highest positivity rate in this group (21/22, 95%—excluding the inconclusive result). Addition of 2ME gives a 100% specificity (24). All AGID testing in this study used the *B. canis* cytoplasmic antigen and had a positivity rate of 95%. AGID is best done 5–10 weeks post-infection, although cytoplasmic antigens can be detected 3 years post-infection (6). AGID sensitivity is reported to decrease with chronicity (26). IFA had a high positivity rate in this study (13/14 dogs, 93%); seroconversion can take up to 8–12 weeks and titer typically declines as bacteremia subsides (24, 38). The variability in positive and negative diagnostic results for *Brucella* serology in our study highlights the need to perform confirmatory tests including culture or PCR, which are not always done routinely.

In previously reported DS studies with imaging, *Staphylococcus* spp. is the most common etiological agent (1–5), and classic radiographic changes include osteolysis of the vertebral endplates and adjacent vertebral bodies, collapse of the intervertebral disc space, variable sclerosis adjacent to lytic regions, variable osseus proliferation adjacent to the space, and occasionally subluxation of the affected disc space (1–3, 19, 33). These changes are generally symmetrical and affect both end-plates cranial and caudal to the intervertebral disc space. Similar changes are described in a prior case series of 14 dogs with BDS (11). While some dogs in our group shared these classical radiographic changes, many also had focal, round, centrally located lesions affecting the intervertebral disc and endplates with no other associated abnormalities (“hole punch” lesions) that might be more unique to BDS. A case report describing multiple hypoattenuating hole-like lesions on CT with early osteolysis on radiographs in a dog with BDS may represent similar lesions to what we described (23). The characteristics of the vertebral end-plate changes did not correlate with duration of clinical signs, indicating that the “hole punch” lesion is not unique to lytic changes early or late in the disease process.

Approximately 2/3 of dogs in this study had multiple lesions on radiographs and MRI compared to previous DS studies of predominately *Staphylococcus* spp. infection where a single lesion was identified in the majority of cases (2, 3, 5, 11). This might be due to the chronic persistence and intermittent shedding of *B. canis* for weeks to years (6, 7, 24, 31, 36), allowing more DS lesions to develop before diagnosis is ultimately made. Although the predisposition for the thoracolumbar and lumbosacral spine was consistent (1–3, 5, 11, 16), approximately 20% of the lesions in these dogs affected the cervical spine, which differs from previous reports where a low number of cervical lesions were identified (1, 2, 5, 11, 16). All DS studies are limited by the lack of complete spinal imaging in many dogs.

MRI features often not apparent on radiographs included vertebral physitis or spondylitis without discitis and paravertebral inflammatory changes without associated disc or vertebral changes. Vertebral physitis has been previously associated with DS in younger dogs, although only one had BDS (33). Paravertebral changes have been described with DS but not as the sole lesion (30). MRI and CT detected changes not evident on radiographs in 21/57 (37%) lesions, and DS would not have been diagnosed based on radiographs alone in one dog. This highlights the importance of advanced imaging in dogs with a clinical history consistent with BDS even if spinal radiographs are normal.

This study has the limitations of a retrospective multi-institutional study where information was not documented in all dogs for all data points. Different *Brucella canis* testing techniques were chosen based on clinician preference, therefore it is difficult to make any conclusions about sensitivity and specificity of the various testing modalities in this group. Complete spinal imaging was not acquired in many cases which skews the location of discospondylitis lesions toward those most clinically evident at time of presentation. The purpose of this retrospective study was to highlight the differences of BDS compared to DS from other bacteria including the younger age at presentation, longer duration of clinical signs, common identification of “hole punch” lesions, and occasional presence of physitis or spondylitis without discitis and paravertebral changes without vertebral or intervertebral disc abnormalities on MRI. *B. canis* may be an emerging pathogen in the southwestern United States and extended blood culture (plated minimum 9 days), *Brucella* PCR, and *Brucella canis* serology should be considered in young dogs presenting with chronic spinal pain even without radiographic evidence of DS. Advanced imaging, in particular STIR MRI imaging, should be pursued in dogs with suspicion of BDS even when spinal radiographs are normal.

## Data availability statement

The original contributions presented in the study are included in the article/Supplementary material, further inquiries can be directed to the corresponding author.

## Author contributions

CL and EB: house officers and manuscript preparation and review. RW: overseeing clinician, study design, manuscript preparation and review, and corresponding author. CC: a board certified radiologist responsible for imaging review. LS, RP, JS, SE, and KK: board certified neurologists who provided cases and assisted with manuscript review. All authors contributed to the article and approved the submitted version.

## Conflict of interest

The authors declare that the research was conducted in the absence of any commercial or financial relationships that could be construed as a potential conflict of interest.

## Publisher's note

All claims expressed in this article are solely those of the authors and do not necessarily represent those of their affiliated organizations, or those of the publisher, the editors and the reviewers. Any product that may be evaluated in this article, or claim that may be made by its manufacturer, is not guaranteed or endorsed by the publisher.

## References

[1] Ruoff CM Kerwin SC Taylor AR. Diagnostic imaging of discospondylitis. Vet Clin North Am Small Anim Pract. (2018) 48:85–94. 10.1016/j.cvsm.2017.08.007 28964545

[2] Carrera I Sullivan M McConnell F Gonçalves R. Magnetic resonance imaging features of discospondylitis in dogs. Vet Radiol Ultrasound. (2011) 52:125–31. 10.1111/j.1740-8261.2010.01756.x 21388462

[3] Burkert BA Kerwin SC Hosgood GL Pechman RD Fontenelle JP. Signalment and clinical features of diskospondylitis in dogs: 513 cases (1980–2001). J Am Vet Med Assoc. (2005) 227:268–75. 10.2460/javma.2005.227.268 16047665

[4] Thomas WB. Diskospondylitis and other vertebral infections. Veterinary Clin North Am Small Anim Practice. (2000) 30:169–82. 10.1016/S0195-5616(00)50008-4 10680214

[5] Harris JM Chen AV Tucker RL Mattoon JS. Clinical features and magnetic resonance imaging characteristics of diskospondylitis in dogs: 23 cases (1997-2010). J Am Vet Med Assoc. (2013) 242:359–65. 10.2460/javma.242.3.359 23327179

[6] Santos RL Souza TD Mol JPS Eckstein C Paíxão TA. Canine brucellosis: an update. Front Vet Sci. (2021) 8:594291. 10.3389/fvets.2021.594291 33738302 PMC7962550

[7] Hensel ME Negron M Arenas-Gamboa AM. Brucellosis in dogs and public health risk. Emerg Infect Dis. (2018) 24:1401–6. 10.3201/eid2408.171171 30014831 PMC6056133

[8] Whitten TV Brayshaw G Patnayak D Alvarez J Larson CM Kustritz MR. Seroprevalence of *Brucella canis* antibodies in dogs entering a Minnesota Humane society, Minnesota, 2016–2017. Prev Vet Med. (2019) 168:90–4. 10.1016/j.prevetmed.2019.04.015 31097129 PMC6592273

[9] Daly R Willis KC Wood J Brown K Brown D Beguin-Strong T. Seroprevalence of *Brucella canis* in dogs rescued from South Dakota Indian reservations, 2015–2019. Prev Vet Med. (2020) 184:105157. 10.1016/j.prevetmed.2020.105157 33002657

[10] Brown J Blue JL Wooley RE Dreesen DW. *Brucella canis* infectivity rates in stray and pet dog populations. Am J Public Health. (1976) 66:889–91. 10.2105/AJPH.66.9.889 961957 PMC1653450

[11] Kerwin SC Lewis DD Hribernik TN Partington B Hosgood G Eilts BE. Diskospondylitis associated with *Brucella canis* infection in dogs: 14 cases (1980–1991). J Am Vet Med Assoc. (1992) 201:1253–7. 1429171

[12] Reynes E López G Ayala SM Hunter GC Lucero NE. Monitoring infected dogs after a canine brucellosis outbreak. Comp Immunol Microbiol Infect Dis. (2012) 35:533–7. 10.1016/j.cimid.2012.05.004 22738948

[13] de Paula Dreer M Gonçalves D da Silva Caetano I Gerônimo E Menegas PH Bergo D. Toxoplasmosis, leptospirosis and brucellosis in stray dogs housed at the shelter in Umuarama municipality, Paraná, Brazil. J Venom Anim Toxins Incl Trop Dis. (2013) 19:23. 10.1186/1678-9199-19-23 24066949 PMC3849923

[14] Greene Craig E Leland E. Carmichael. Canine brucellosis, infectious diseases of the dog and cat, Fourth Edition. (2012). [EChapter 38].

[15] Buhmann G Paul F Herbst W Melzer F Wolf G Hartmann K. Canine brucellosis: insights into the epidemiologic situation in Europe. Front Vet Sci. (2019) 6:151. 10.3389/fvets.2019.00151 31214601 PMC6554662

[16] Davis MJ Dewey CW Walker MA Kerwin SC Moon ML Kortz GD. Contrast radiographic findings in canine bacterial discospondylitis: a multicenter, retrospective study of 27 cases. J Am Anim Hosp Assoc. (2000) 36:81–5. 10.5326/15473317-36-1-81 10667411

[17] Canal S Contiero B Balducci F Calò P Bernardini M. Risk factors for diskospondylitis in dogs after spinal decompression surgery for intervertebral disk herniation. J Am Vet Med Assoc. (2016) 248:1383–90. 10.2460/javma.248.12.1383 27270060

[18] Kornegay JN Barber DL. Diskospondylitis in dogs. J Am Vet Med Assoc. (1980) 177:337–41. 7451303

[19] Kirberger RM. Early diagnostic imaging findings in juvenile dogs with presumed diskospondylitis: 10 cases (2008–2014). J Am Vet Med Assoc. (2016) 249:539–46. 10.2460/javma.249.5.539 27556268

[20] Trub SA Bush WW Paek M Cuff DE. Use of C-reactive protein concentration in evaluation of diskospondylitis in dogs. J Vet Intern Med. (2021) 35:209–16. 10.1111/jvim.15981 33319417 PMC7848344

[21] Fischer A Mahaffey MB Oliver JE. Fluoroscopically guided percutaneous disk aspiration in 10 dogs with diskospondylitis. J Veter Intern Med. (1997) 11:284–7. 10.1111/j.1939-1676.1997.tb00466.x 9348495

[22] Kornegay JN. Diskospondylitis. In: Kirk RW., Ed. Current Veterinary Therapy VII. W. B. Saunders Co. (1983):718–722.

[23] Forbes JN Frederick SW Savage MY Cross AR. *Brucella canis* sacroiliitis and discospondylitis in a dog. Can Vet J. (2019) 60:1301–4. 31814636 PMC6855227

[24] Cosford KL. *Brucella canis*: an update on research and clinical management. Can Vet J. (2018) 59:74–81. 29302106 PMC5731389

[25] Gomes SA Garosi LS Behr S Toni C Tabanez J Rusbridge C. Clinical features, treatment and outcome of discospondylitis in cats. J Feline Med Surg. (2022) 24:311–21. 10.1177/1098612X211020159 34100660 PMC10812237

[26] Mol JPS Guedes ACB Eckstein C Quintal AP Souza TD Mathias LA. Diagnosis of canine brucellosis: comparison of various serologic tests and PCR. J Vet Diagn Invest. (2020) 32:77–86. 10.1177/1040638719891083 31752635 PMC7003229

[27] Cedraschi C Robert J Goerg D Perrin E Fischer W Vischer TL. Is chronic non-specific low back pain chronic? Definitions of a problem and problems of a definition. Br J Gen Pract. (1999) 49:358–62. 10736885 PMC1313420

[28] Fahie MA Cortez JC Ledesma M Su Y. Pressure mat analysis of walk and trot gait characteristics in 66 normal small, medium, large, and giant breed dogs. Front Vet Sci. (2018) 5:256. 10.3389/fvets.2018.00256 30386786 PMC6198868

[29] Jolivet F Pic M Rishniw M Concordet D Dossin O. The use of thermometer protective sheets provides reliable measurement of rectal temperature: a prospective study in 500 dogs. J Small Anim Pract. (2020) 61:216–23. 10.1111/jsap.13119 32065392

[30] Gonzalo-Orden JM Altónaga JR Orden MA Gonzalo JM. Magnetic resonance, computed tomographic and radiologic findings in a dog with discospondylitis. Vet Radiol Ultrasound. (2000) 41:142–4. 10.1111/j.1740-8261.2000.tb01467.x 10779073

[31] Hurov L Troy G Turnwald G. Diskospondylitis in the dog: 27 cases. J Am Vet Med Assoc. (1978) 173:275–81. 689970

[32] Gilmore DR. Lumbosacral discospondylitis in 21 dogs. J Am Anim Hosp Assoc. (1987) (23):57–61.

[33] Jimenez MM O'Callaghan MW VERTEBRAL PHYSITIS. A RADIOGRAPHIC DIAGNOSIS TO BE SEPARATED FROM DISCOSPONDYLITIS. A Prelim Rep Veter Radiol Ultrasound. (1995) 36:188–95. 10.1111/j.1740-8261.1995.tb00245.x

[34] Keid LB Soares RM Vasconcellos SA Megid J Salgado VR Richtzenhain LJ. Comparison of agar gel immunodiffusion test, rapid slide agglutination test, microbiological culture and PCR for the diagnosis of canine brucellosis. Res Vet Sci. (2009) 86:22–6. 10.1016/j.rvsc.2008.05.012 18656213

[35] Flores-Castro R Segura R A. serological and bacteriological survey of canine brucellosis in Mexico. Cornell Vet. (1976) 66:347–52. 954441

[36] Carmichael LE Zoha SJ Flores-Castro R. Problems in the serodiagnosis of canine brucellosis: dog responses to cell wall and internal antigens of *Brucella canis*. Dev Biol Stand. (1984) 56:371–83. 6489621

[37] Hayoun MA Muco E Shorman M. Brucellosis. In: StatPearls. StatPearls Publishing. (2022). (accessed May 9, 2022). Available online at: http://www.ncbi.nlm.nih.gov/books/NBK441831/28722861

[38] Johnson CA Carter TD Dunn JR Baer SR Schalow MM Bellay YM. Investigation and characterization of *Brucella canis* infections in pet-quality dogs and associated human exposures during a 2007–2016 outbreak in Michigan. J Am Vet Med Assoc. (2018) 253:322–36. 10.2460/javma.253.3.322 30020006 PMC6642745

[39] Lorenz MD Coates JR Kent M. Pelvic limb paresis, paralysis, or ataxia. In: Handbook of Veterinary Neurology. 5th Ed. Elsevier Saunders. (2011):124–6. 10.1016/B978-1-4377-0651-2.10006-2

[40] Huggins C THE PHYSIOLOGY OF THE PROSTATE. GLAND. Physiol Rev. (1945) 25:281–95. 10.1152/physrev.1945.25.2.281

