# Supplementary material for: Brucella canis discospondylitis in 33 dogs
Source: Front Vet Sci. 2022 Nov 4;9:1043610. doi: 10.3389/fvets.2022.1043610 (PMC9672337; doi:10.3389/fvets.2022.1043610)
Supplement: Supplementary file 1 [file Data_Sheet_1.docx]

**Table 1. Signalment, duration of clinical signs, origin**

| **Patient** | **Breed** | **Sex** | **Age (years)** | **Weight (Kgs)** | **Current residence** | **Origin of Adoption** | **Duration of Clinical Signs** |
| --- | --- | --- | --- | --- | --- | --- | --- |
| 1 | German Shepherd Mix | FS | 4.5 | 27.9 | Arizona | New Mexico | 6 months |
| 2 | Husky Mix | MN | 10 | 26.1 | Arizona | Arizona | 3 months |
| 3 | Bernese Mountain Dog | MN | 6 | 43.6 | Arizona | NR | 5 weeks |
| 4 | Australian Cattle Dog Mix | MN | 1.16 | 26.1 | Arizona | Arizona | 7 months |
| 5 | German Shepherd | MN | 3 | 38.2 | Arizona | Arizona | 3 months |
| 6 | Mixed Breed Dog | FS | 1.41 | 19.2 | Arizona | Arizona | 3 weeks |
| 7 | Labrador Retriever | FI | 0.9 | 22.4 | Arizona | Arizona | 9 months |
| 8 | Shepherd Mix | FS | 4.3 | 25.3 | Arizona | Arizona | NR* |
| 9 | Shepherd Mix | FS | 3 | 39 | Arizona | Mexico | 4 months |
| 10 | Border Collie Mix | MN | 1.7 | 13.1 | Colorado | Mexico | 1 week* |
| 11 | Chow Chow Mix | MN | 2 | 31.2 | Colorado | Texas | 1 year |
| 12 | Labrador Retriever Mix | MN | 2 | 24.9 | Colorado | Texas | Weeks |
| 13 | Australian Shepherd Mix | MN | 2 | 43 | Colorado | Indian reservation | 6 months |
| 14 | Mini Australian Shepherd | MN | 1 | 15.8 | Colorado | Nebraska | 6 months |
| 15 | German Shepherd Mix | MN | 2 | 24.3 | Colorado | NR | 2 months |
| 16 | Shepherd Mix | MN | 4 | 28.6 | Colorado | Texas | 2 months |
| 17 | Mixed Breed Dog | MN | 3 | 37.2 | Colorado | NR | 9 days* |
| 18 | Chow Chow Mix | MN | 3 | 31.6 | Colorado | Texas | 1-2 years |
| 19 | Mixed Breed Dog | FS | 4 | 27 | Colorado | New Mexico | 4 years |
| 20 | Mixed Breed Dog | MN | 2 | 28.4 | Colorado | NR | 1 year |
| 21 | Labrador Retriever | MN | 4.5 | 27 | Colorado | Texas | 5 months |
| 22 | Staffordshire Terrier Mix | FS | 4 | 26.2 | Colorado | New Mexico | 24 months |
| 23 | Labarador Retriever | FS | 3 | 23.7 | Colorado | Colorado | 30 months |
| 24 | German Shepherd Mix | FS | 0.83 | 12.8 | Colorado | NR | 7 months |
| 25 | Shepherd Mix | MN | 1.5 | 18 | Colorado | New Mexico | 6 months |
| 26 | Mixed Breed Dog | MN | 2.3 | 31.4 | Colorado | Texas | NR |
| 27 | Mixed Breed Dog | MN | 0.9 | 15 | Colorado | Texas | 7 months |
| 28 | Mixed Breed Dog | MN | 4 | 33.4 | Colorado | NR | 5 days |
| 29 | Mixed Breed Dog | MN | 2.5 | 30.1 | Colorado | NR | NR |
| 30 | Labrador Retriever | MN | 1.5 | 37.2 | Colorado | Colorado | 3 months |
| 31 | Mixed Breed Dog | FS | 6 | 23.8 | Colorado | NR | NR |
| 32 | Mixed Breed Dog | MN | 2.5 | 8.65 | Colorado | NR | Several months |
| 33 | Border Collie Mix | FS | 1.5 | 50.5 | Colorado | NR | 6 months |

*Previous history of spinal pain 1 year prior

**Table 2. Temperature, CBC, and Biochemistry Results**

| **Patient** | | **Temperature (F)** | | **CBC (cells/uL)** | | **Chemistry** | |
| --- | --- | --- | --- | --- | --- | --- | --- |
| 1 | | 100.4 | | Lymphopenia (620) Neutrophilia (14900) | | WNL | |
| 2 | | 102 | | WNL | | WNL | |
| 3 | | 103.7 | | WNL | | Hyperglobulinemia (4.8) ALP elevation (219) | |
| 4 | | 101.7 | | NR | | NR | |
| 5 | | 102.6 | | WNL | | WNL | |
| 6 | | 101.1 | | WNL | | Hyperglobulinemia (4.6) | |
| 7 | | 102.8 | | NR | | NR | |
| 8 | | 101.5 | | WNL | | Lipase elevation (813) | |
| 9 | | 102 | | WNL | | WNL | |
| 10 | | 100.4 | | WNL | | WNL | |
| 11 | | 100.4 | | Monocytopenia (71) | | Hyperproteinemia (7.4)  Hyperglobulinemia (3.9) Hyperbilirubinemia (0.5) | |
| 12 | | 101.3 | | NR | | NR | |
| 13 | | 102.4 | | NR | | NR | |
| 14 | | 100.9 | | WNL | | WNL | |
| 15 | | 99.4 | | NR | | NR | |
| 16 | | 99.7 | | NR | | NR | |
| 17 | | 101.9 | | NR | | NR | |
| 18 | | 101.3 | | Thrombocytopenia (148,000) | | NR | |
| 19 | | NR | | NR | | NR | |
| 20 | | 101.5 | | WNL | | WNL | |
| 21 | | 101.1 | | WNL | | WNL | |
| 22 | | 100.9 | | WNL | | WNL | |
| 23 | | 101.3 | | Neutrophilia (10,603) | | ALP elevation (435) | |
| 24 | | 101.5 | | WNL | | WNL | |
| 25 | | 100.8 | | WNL | | WNL | |
| 26 | | 101.7 | | WNL | | Hyperphosphatemia (7.4) | |
| 27 | | 101.9 | | NR | | NR | |
| 28 | | 102.3 | | NR | | NR | |
| 29 | | NR | | NR | | NR | |
| 30 | | 102.7 | | NR | | NR | |
| 31 | | NR | | NR | | NR | |
| 32 | | NR | | NR | | NR | |
| 33 | | NR | | NR | | NR | |
|  | | |  |  |  |  |  |
|  |  | | | |  | |  |

WNL=Within normal limits; NR=not reported

**Table 3: Brucella Diagnostics**

| Patient | Zoetis D-Tec 2-ME RSAT | Brucella IFA | Brucella AGID | Cornell 2-ME RSAT | Urine Culture | Blood Culture | Aspergillus Antigen | Coccidioides Antibody |
| --- | --- | --- | --- | --- | --- | --- | --- | --- |
| 1 | NR | 1:1600 | NR | NR | Negative | Positive for Brucella canis | Negative | Negative |
| 2 | NR | 1:3200 | NR | NR | Negative | Negative | NR | Negative |
| 3 | NR | 1:200 | NR | NR | NR | Negative | Negative | Negative |
| 4 | NR | 1:2800 | NR | NR | Negative | Positive for Brucella canis | NR | NR |
| 5 | NR | 1:800 | NR | NR | Negative | Negative | NR | Negative |
| 6 | NR | Negative | Negative | Positive | Negative | Positive for Brucella canis | NR | NR |
| 7 | NR | 1:3200 | NR | NR | Negative | Positive for Brucella canis | NR | Negative |
| 8 | NR | NR | Positive | Positive | NR | NR | Negative | Negative |
| 9 | NR | 1:2800 | Positive | Positive | Negative | Positive for Brucella canis | NR | NR |
| 10 | Positive | NR | Positive | Positive | NR | NR | NR | Negative |
| 11 | Positive | NR | Positive | Positive | NR | NR | Negative | Negative |
| 12 | Positive | NR | Suspicious | Positive | NR | NR | NR | NR |
| 13 | NR | NR | NR | NR | Negative | Positive for Brucella canis | NR | NR |
| 14 | NR | NR | NR | Positive | Positive for Brucella | Positive for Brucella canis | NR | NR |
| 15 | NR | NR | NR | Positive | Negative | Positive for Brucella canis | NR | NR |
| 16 | NR | >1:50 | Positive | Positive | NR | Negative | NR | NR |
| 17 | NR | NR | NR | Positive | NR | NR | NR | NR |
| 18 | Positive | NR | Positive | NR | NR | NR | NR | NR |
| 19 | Positive | NR | Positive | Positive | NR | NR | NR | NR |
| 20 | Positive | NR | Positive | NR | NR | NR | NR | NR |
| 21 | Inconclusive | NR | Positive | NR | NR | Negative | NR | NR |
| 22 | NR | NR | Positive | NR | NR | NR | NR | NR |
| 23 | NR | NR | Positive | NR | NR | NR | NR | NR |
| 24 | NR | >1:50 | Positive | NR | Negative | NR | NR | NR |
| 25 | NR | >1:50 | Positive | NR | Negative | NR | NR | NR |
| 26 | NR | >1:50 | Positive | NR | Negative | NR | NR | NR |
| 27 | NR | NR | Positive | Positive | Negative | Positive for Brucella canis | NR | NR |
| 28 | NR | NR | Positive | Positive | Negative | Positive for Brucella canis | NR | NR |
| 29 | NR | NR | Positive | NR | NR | NR | Negative | NR |
| 30 | NR | >1:200 | Positive | NR | Negative | Positive for Brucella canis | NR | NR |
| 31 | NR | 1:3200 | NR | NR | NR | NR | NR | NR |
| 32 | NR | NR | Positive | Positive | NR | Positive for Brucella canis | NR | NR |
| 33 | NR | NR | NR | Positive | NR | Positive for Brucella canis | NR | NR |
